# Diagnostic performance of PSMA PET/CT, multiparametric MRI, and combined imaging for local prostate cancer recurrence after radical prostatectomy

**DOI:** 10.1007/s00261-025-05256-5

**Published:** 2025-11-08

**Authors:** Michael Phillipi, David Choung, Erwin Ho, Samuel Anavim, Shayan Saeed, Chang Shu, James Shi, Justin Glavis-Bloom, Arti Gupta, Akash Joshi, Amir Imanzadeh, Steven Seyedin, Edward Uchio, Michael Daneshvar, Roozbeh Houshyar

**Affiliations:** 1 Department of Radiological Sciences, University of California, Irvine, Irvine, USA; 2 Department of Radiation Oncology, University of California, San Francisco, San Francisco, USA; 3 Department of Urology, University of California, Irvine, Irvine, USA

**Keywords:** Prostate cancer, Multiparametric MRI, PSMA PET/CT, Prostatectomy, Local recurrence

## Abstract

**Objectives:**

Approximately 20–50% of patients develop biochemical recurrence (BCR) of prostate cancer within 10 years following radical prostatectomy (RP). The accurate identification of recurrent disease is crucial for guiding salvage treatment decisions. While multiparametric MRI (mpMRI) and prostate-specific membrane antigen positron emission tomography/computed tomography (PSMA PET/CT) are both utilized for detecting local recurrence, their combined diagnostic benefits remain unclear. This study seeks to evaluate the diagnostic performance of both modalities alone and in conjunction for detecting local recurrence following RP in patients with BCR.

**Methods:**

A retrospective single-institution analysis included 37 post-RP patients with BCR who received mpMRI and PSMA PET/CT. Five board-certified radiologists reviewed images in three phases: mpMRI only, PSMA PET/CT only, and both modalities combined. Multidisciplinary tumor board consensus served as the reference standard. Diagnostic performance, inter-reader agreement, and radiologist confidence with each modality was examined.

**Results:**

MpMRI outperformed PSMA PET/CT, yielding a higher sensitivity (73.0% vs. 65.2%) and specificity (77.1% vs. 75.7%). Interpretation of mpMRI and PSMA PET/CT together achieved the highest diagnostic accuracy (77.8%), representing a statistically-significant increase over PSMA PET/CT (*p* = 0.026) but a non-statistically-significant increase over mpMRI (*p* = 0.441). Combined imaging also resulted in greater specificity (90.0%) and inter-rater reliability (κ = 0.622). However, in some cases performance decreased with both modalities due to interpretive pitfalls.

**Conclusion:**

While mpMRI remains the preferred imaging modality for post-RP local recurrence surveillance, the integration of PSMA PET/CT may lead to improved specificity and inter-rater reliability. However, radiologists must understand each modality’s limitations to avoid interpretive pitfalls.

## Introduction

Prostate cancer is the second most common cause of male cancer worldwide, with 1.4 million new cases diagnosed annually [1]. Approximately 40% of patients with localized prostate cancer will elect to undergo the curative treatment of radical prostatectomy (RP), which removes both the prostate gland and seminal vesicles [2]. However, 20 to 50% of men will develop biochemical recurrence (BCR) of cancer within 10 years following initial curative treatment, defined as a consecutive rising serum prostatic-specific antigen (PSA) or PSA greater than 0.2 ng/ml [3, 4]. The accurate localization of the recurrence of prostate cancer is an important first step to guiding salvage treatment decisions, such as radiation therapy, androgen deprivation therapy (ADT), early systemic chemotherapy, or combinations of these treatments. Early identification of local recurrence impacts both radiation targeting and dose to the gross disease [5].

Traditionally, conventional imaging modalities such as computed tomography (CT) and ultrasound have been utilized in both the initial staging and restaging of prostate cancer recurrence in patients with BCR [6]. In recent years, multiparametric magnetic resonance imaging (mpMRI) has been increasingly adopted in this clinical setting. MpMRI consists of T2-weighted imaging (T2WI), diffusion-weight imaging (DWI), and dynamic contrast-enhanced imaging (DCE), which supplies interpreting radiologists with both anatomical and functional information regarding the prostate and prostate bed [7]. However, following RP the prostate bed undergoes substantial anatomical and structural modifications, including fibrosis and scarring, complicating radiological assessment for recurrent disease [8]. Further, while signal characteristics of the recurrent tumors are commonly similar to the initial tumor, the lack of surrounding normal prostatic tissue as a comparative baseline, coupled with the altered post-operative landscape, can make definitive diagnosis particularly challenging [2].

Meanwhile, prostate-specific membrane antigen positron emission tomography computed tomography (PSMA PET/CT) has been increasingly utilized clinically for the superior detection of disease in the primary and recurrent prostate cancer setting [9]. Although current guidelines endorse the use of PSMA imaging for prostate cancer patients in a variety of clinical scenarios, including in the setting of BCR after RP, its efficacy in detecting local recurrence remains unclear [10]. In clinical practice, PSMA PET/CT is predominantly utilized for metastatic disease detection rather than local recurrence identification [11]. The close proximity of the vesicourethral anastomosis to the bladder after RP can cause physiological urinary extraction of PSMA tracers to create artifacts, obscuring the detection of local recurrence [12].

Several studies suggest that mpMRI may provide superior imaging sensitivity, specificity, and accuracy for detecting prostate bed recurrence compared to PSMA PET/CT [13]. However, to our knowledge the potential synergistic combination of PSMA PET/CT and MRI for the detection of prostate bed recurrence after RP has not been extensively studied. Given the effect of accurate and early detection on treatment planning, it is essential to establish evidence-based imaging protocols for this clinical scenario [14]. In this study, we aim to evaluate the diagnostic performance of PSMA PET/CT alone, mpMRI alone, and both imaging techniques in conjunction for detecting the local recurrence of prostate cancer following RP. Through a comparison of the overall accuracy, sensitivity, and specificity, in addition to the inter-reader reliability and intra-reader agreement between board-certified abdominal radiologists, we seek to provide further evidence towards the optimal imaging sequence in post-RP BCR surveillance.

## Materials and methods

### Cohort composition

This retrospective cohort study of post-prostatectomy patients was conducted at a single institution in Southern California and was approved by the Institutional Review Board (HS#2015-2204). Patients with a history of prostate cancer treated with prostatectomy were identified through a query of cases discussed at our institution’s tumor board. Patient charts were then reviewed for imaging completed after treatment. Patients that did not receive both mpMRI and PSMA PET/CT imaging were excluded. Patients were included irrespective of whether mpMRI or PSMA PET/CT was the initial study, as imaging order was determined by individual clinical indications. Similarly, no exclusion criteria were applied for prior treatments before radical prostatectomy, as the impact of pre-operative treatments on post-surgical prostate bed imaging interpretation remains poorly characterized. Given the absence of histopathologic examination in these patients because of the inability to biopsy most recurrence due to small size, patients were identified as positive or negative for local recurrence based on an imaging review by a combination of expert radiologists, urologists, medical oncologists and radiation oncologists at a genitourinary tumor board meeting [15]. Patients both positive and negative for the local recurrence of prostate cancer were included.

### MpMRI

MpMRIs were performed on a 3.0 Tesla Siemens Magnetom Vida MRI unit (Siemens Healthcare, Erlangen, Germany) without the use of an endorectal coil. Imaging sequences adhered to PI-RADS v2.1 standards and included: T1 axial whole pelvis, T2 sagittal, coronal, and axial high-resolution small field-of-view imaging, axial proton-density high-resolution fast spin-echo, axial DWI with b-values of 50, 400, and > 1400 s/mm², ADC maps, dynamic contrast-enhanced imaging, and T1 axial fat-saturated post-contrast large field-of-view imaging.

### PSMA PET/CT

PSMA PET/CT scans were acquired using a GE Advance PET/CT scanner. Patients were intravenously injected with 18 F-rhPSMA, 18 F-piflufolastat, 18 F-Flotufolastat or 68Ga–PSMA-11, followed by an uptake period of approximately one hour prior to image acquisition. PET imaging was performed from the base of the skull to the mid-thigh. Images were reconstructed into attenuation corrected tomographic slices in the transaxial, coronal, and sagittal projections. The CT portion was performed with a low-dose technique without intravenous contrast for image fusion, localization, and attenuation correction. Imaging adhered to standardized acquisition and processing protocols consistent with NEMA XR-29 safety standards.

### Imaging review

Five board-certified abdominal radiologists independently reviewed imaging in three phases. The participating radiologists each have approximately seven years of experience interpreting approximately 300 to 400 prostate MRI or PSMA PET/CT scans annually on average, with about 10% of those cases involving post-prostatectomy patients. These participants were asked to review patient imaging in three separate phases. For each case, only MRI images were reviewed in the first phase, while PSMA PET/CT images were reviewed in the second. During the third phase, both imaging modalities were reviewed in conjunction. Each phase was conducted one month apart to minimize recall. For each case in each phase, reviewers were prompted to note if they believed there was local recurrence of cancer. Reviewers were also prompted to rate their confidence in their assessment on a 1 to 5 scale. A score of 1 was described as “extremely not confident,” 2 was “somewhat not confident,” 3 was “neutral,” 4 was “somewhat confident,” and 5 was “extremely confident.”

### Statistical analysis

Reviewer responses were compared to the Tumor Board consensus, which was considered the reference standard. Statistical analysis was performed using NumPy 2.20 and SciPy v1.15.3. The accuracy, sensitivity, specificity, positive and negative predictive value (PPV, NPV) were calculated to compare diagnostic performance. Diagnostic accuracy was defined as the number of cases in which the reviewer correctly identified either positive or negative local recurrence, divided by the total number of cases examined. Fleiss’ kappa scores were used to determine inter-rater reliability, while Quadratic weighted Cohen’s kappa and Kendall’s Tau coefficients were used to assess intra-reader agreement. Inter-reliability and intra-reader agreement was assessed based on the presence or absence of recurrence. Reader confidence between modalities was analyzed using descriptive statistics. McNemar’s test was used to compare paired proportions between modalities. Fisher’s exact test was used to compare independent groups in a subanalysis. Two-tailed p values of less than 0.05 were considered statistically significant.

## Results

### Demographic and clinical details of the cohort

Thirty-seven patients completed both mpMRI and PSMA PET/CT imaging after RP for the treatment of prostate cancer from December 2020 to March 2024 were qualified for this study with a mean age of 71. PSMA PET/CT and mpMRI imaging were performed at an average of 65.3 days apart, and initial imaging was performed at an average of 5.35 years after RP. Mean and median PSA levels with closest proximity to the date of initial imaging were 3.7 and 0.7, respectively. PSMA PET/CT was performed with 18 F-rhPSMA for two subjects, 18 F-piflufolastat for 20 subjects, 18 F-Flotufolastat for one subject and 68Ga–PSMA-11 for 14 subjects. One subject’s lesion was smaller than 5 mm, while ten had lesions 5 mm to 10 mm in size, and twelve had lesions greater than 10 mm. Prior to imaging, six patients had received salvage radiation treatment, while fifteen patients had received androgen deprivation therapy. Twenty-three (62.2%) cases were considered positive for the local recurrence of prostate cancer through a case review at our institution’s genitourinary tumor board. This review also determined that five of these patients had multiple lesions, while four subjects had a lesion involving the bladder. Full demographic and clinical details of the cohort are described in Table 1.

**Table 1:** Overview of subject History, imaging details and recurrence characteristics

Subject details		Primary tumor characteristics and treatment					Imaging information			Recurrence characteristics		
Subject	Age (years)	Path- ological stage	Gleason score	Radiation therapy	Brachy-therapy	ADT	PSA level (ng/mL)	Time between imaging (Days)	PET study type	Lesion size (mm)	Involving bladder	Multiple lesions
1	60	T3a	3 + 4 = 7	No	No	No	0.2	36	F18	NA	NA	NA
2	68	T3a	3 + 4 = 7	No	No	No	1.54	25	Ga68	> 10	No	No
3	85	T2	3 + 4 = 7	No	No	No	3.1	82	F18	5–10	No	No
4	65	T2	3 + 4 = 7	No	No	No	0.07	129	Ga68	NA	NA	NA
5	77	T2	3 + 4 = 7	No	No	Yes	0.59	3	F18	NA	NA	NA
6	79	NA	4 + 5 = 9	No	No	Yes	1.27	484	F18	5–10	No	No
7	75	T3	3 + 4 = 7	No	No	No	0.23	6	F18	NA	NA	NA
8	69	T2c	3 + 4 = 7	No	No	No	2.4	7	F18	5–10	No	No
9	73	T3	3 + 4 = 7	No	No	No	0.7	1	Ga68	NA	NA	NA
10	68	T3b	4 + 3 = 7	No	No	No	1.16	9	F18	NA	NA	NA
11	76	T2c	4 + 4 = 8	Yes	No	Yes	3.47	33	Ga68	> 10	No	No
12	71	T3a	4 + 3 = 7	No	No	No	0.5	13	Ga68	NA	NA	NA
13	81	T3a	4 + 5 = 9	No	No	No	1.3	1	F18	5–10	No	No
14	64	T3	4 + 4 = 8	No	No	No	0.23	2	F18	NA	NA	NA
15	67	T3a	5 + 4 = 9	No	No	Yes	0.2	29	Ga68	NA	NA	NA
16	70	T2	5 + 4 = 9	No	No	Yes	29.5	10	Ga68	> 10	Yes	No
17	66	T2	4 + 3 = 7	No	No	Yes	0.48	21	F18	< 5	No	No
18	78	T3b	4 + 4 = 8	Yes	No	No	2.4	3	Ga68	5–10	No	Yes
19	61	T3b	5 + 4 = 9	Yes	No	No	4.8	9	Ga68	> 10	No	No
20	79	T2	3 + 4 = 7	No	No	No	0.28	13	Ga68	5–10	No	No
21	64	T2b	4 + 3 = 7	No	No	No	1	22	F18	5–10	No	No
22	53	T3b	4 + 5 = 9	No	No	No	0.2	5	F18	5–10	Yes	No
23	75	T3a	4 + 5 = 9	No	No	No	2.34	140	Ga68	> 10	No	No
24	49	T3b	5 + 4 = 9	No	No	No	0.1	4	Ga68	> 10	No	Yes
25	67	T3a	3 + 4 = 7	Yes	No	Yes	0.01	166	F18	5–10	No	No
26	69	T3b	4 + 3 = 7	No	No	No	2.59	13	Ga68	> 10	No	No
27	82	T3a	3 + 4 = 7	No	No	Yes	2.5	463	F18	> 10	No	No
28	86	NA	NA	No	No	Yes	0.5	173	F18	> 10	Yes	Yes
29	77	T2	3 + 4 = 7	No	No	Yes	0.1	10	F18	> 10	No	Yes
30	70	T3a	3 + 4 = 7	No	No	Yes	0.46	179	F18	NA	NA	NA
31	64	T3a	3 + 3 = 6	Yes	No	Yes	0.01	12	F18	NA	NA	NA
32	81	T2	4 + 5 = 9	No	No	No	0.39	43	F18	NA	NA	NA
33	65	T3a	4 + 4 = 8	No	No	No	0.86	3	Ga68	NA	NA	NA
34	86	T2	3 + 4 = 7	Yes	No	Yes	1.8	19	F18	NA	NA	NA
35	57	T3b	5 + 4 = 9	No	No	No	0.4	23	F18	5–10	No	No
36	81	NA	NA	No	No	Yes	64.7	105	F18	> 10	Yes	No
37	75	T3b	4 + 4 = 8	No	No	Yes	5.9	121	F18	> 10	No	Yes
Mean	71.2	NA	NA	NA	NA	NA	3.7	65.3	NA	NA	NA	NA
Median	70.0	NA	NA	NA	NA	NA	0.7	19.0	NA	NA	NA	NA

*ADT* androgen deprivation therapy, *PSA* = Prostate-specific antigen, *F18* Fluorine-18-PSMA, *Ga-68* Gallium-Ga 68 PSMA-11

## Performance

**Fig. 1:**
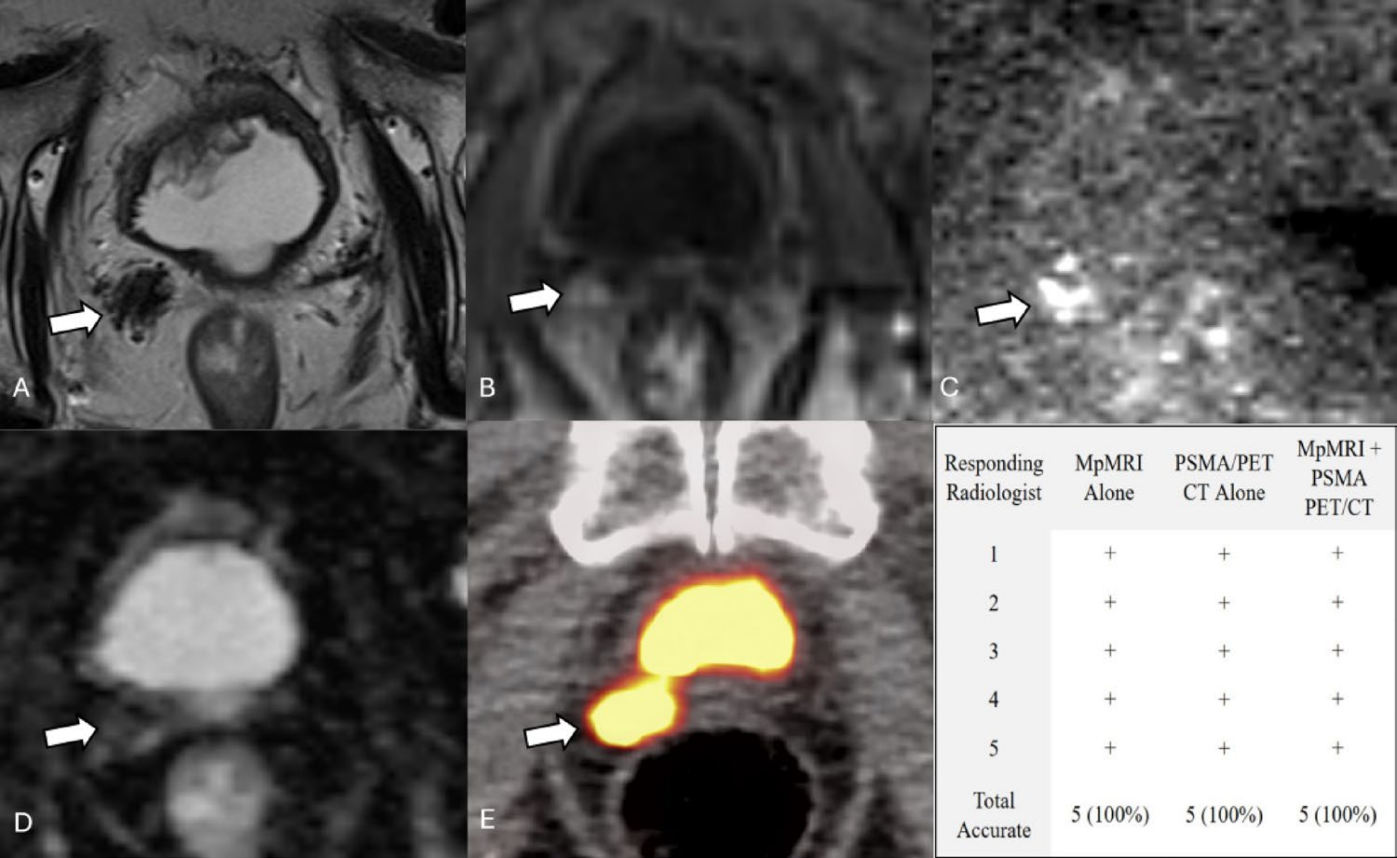
86-year-old patient with a right prostate bed lesion with focal uptake on PSMA PET/CT and focal abnormalities on mpMRI This case depicts an 86-year-old status-post prostatectomy indicated for a primary adenocarcinoma. His PSA was measured to be 0.5 ng/mL at the time of imaging. MpMRI (**A** T2, **B** Arterial enhancement T1, **C** DWI, **D** ADC) shows a > 10 mm right prostate bed focal T2 dark lesion with arterial hyperenhancement and restricted diffusion (white arrows). 18 F-piflufolastat PSMA PET/CT (**E**) shows focal uptake in the same region of the right prostate bed (white arrow). MpMRI and PSMA PET/CT were performed 173 days apart. In this case, performance was identical with mpMRI alone, PSMA PET/CT alone, and both modalities in conjunction (**F**)

With mpMRI, radiologists had an accuracy of 74.6%, a sensitivity of 73.0%, a specificity of 77.1%, a PPV of 84.0%, and a NPV of 63.5% (Table 2). With PSMA PET/CT, the diagnostic accuracy decreased to 69.2% (*p* = 0.275). PSMA PET/CT also had a lower sensitivity (65.2%), specificity (75.7%), PPV (81.5%) and NPV (57.0%). The use of both imaging modalities led to a statistically significant increase in diagnostic accuracy (77.8%) when compared to PSMA PET/CT (*p* = 0.026) and a non-statistically significant increase when compared to mpMRI (*p* = 0.441). The highest specificity (90.0%), PPV (92.1%), and NPV (66.0%) were achieved when using both imaging modalities together. Figure 1 demonstrates a representative case where local recurrence was correctly identified on both mpMRI and PSMA PET/CT, illustrating the complementary information provided by each modality.

**Table 2:** Radiologist diagnostic performance

Radiologist	Accuracy (%)	Sensitivity (%)	Specificity (%)	PPV (%)	NPV (%)	Modality
Attending 1	67.6	52.2	92.9	92.3	54.2	MpMRI
Attending 2	70.3	87.0	42.9	71.4	66.7	MpMRI
Attending 3	78.4	91.3	57.1	77.8	80.0	MpMRI
Attending 4	86.5	78.3	100.0	100.0	73.7	MpMRI
Attending 5	70.3	56.5	92.9	92.9	56.5	MpMRI
**Average**	**74.6 (*p*** = 0.275*)	**73.0**	**77.1**	**84.0**	**63.5**	**MpMRI**
Attending 1	67.6	52.2	92.9	92.3	54.2	PSMA PET/CT
Attending 2	73.0	82.6	57.1	76.0	66.7	PSMA PET/CT
Attending 3	62.2	69.6	50.0	69.6	50.0	PSMA PET/CT
Attending 4	70.3	56.5	92.9	92.9	56.5	PSMA PET/CT
Attending 5	73.0	65.2	85.7	88.2	60.0	PSMA PET/CT
**Average**	**69.2 (*p*** = 0.026**)	**65.2**	**75.7**	**81.5**	**57.0**	**PSMA**
Attending 1	70.3	56.5	92.9	92.9	56.5	MpMRI + PSMA PET/CT
Attending 2	83.8	78.3	92.9	94.7	72.2	MpMRI + PSMA PET/CT
Attending 3	83.8	78.3	92.9	94.7	72.2	MpMRI + PSMA PET/CT
Attending 4	81.1	73.9	92.9	94.4	68.4	MpMRI + PSMA PET/CT
Attending 5	70.3	65.2	78.6	83.3	57.9	MpMRI + PSMA PET/CT
Average	**77.8 (*p*** = 0.441***)	**70.4**	**90.0**	**92.1**	**66.0**	**MpMRI + PSMA PET/CT**

*mpMRI versus PSMA PET/CT p-value, **PSMA PET/CT versus PSMA PET/CT + mpMRI p-value, ***mpMRI versus PSMA PET/CT + mpMRI p-value

Radiologists using both imaging methods in conjunction had the greatest average inter-rater reliability (κ = 0.621, substantial agreement versus 0.438, moderate agreement for PSMA PET/CT versus 0.391, fair agreement for mpMRI). Average intra-reader agreement was the greatest when comparing results from the use of mpMRI alone and PSMA PET/CT alone to results from both modalities in conjunction (κ = 0.536, τ = 0.560 and κ = 0.497, τ = 0.506, respectively). Intra-reader agreement between PSMA PET/CT and MRI was lower (κ = 0.188, τ = 0.191). Individual radiologist performance varied. Of the five participating radiologists, two had a higher diagnostic accuracy with mpMRI than with PSMA PET/CT, while two were more accurate with PSMA PET/CT, and one radiologist had identical accuracy with both modalities. Three radiologists yielded the highest personal accuracy when using both PSMA PET/CT and mpMRI imaging. However, the performance of two of the five participating radiologists declined with the use of both modalities in comparison to their highest accuracy with either mpMRI or PSMA PET/CT.

### Subgroup analyses

Subgroup analyses were conducted on patients with multiple lesions within the prostate bed, lesions involving the urinary bladder, lesions grouped by size, patients who received pre-prostatectomy radiation treatment, and PSMA PET/CT diagnostic agent administered (Table 3). When examining the five patients with multiple lesions within the prostate bed, diagnostic accuracy with mpMRI alone was 88.0% while it was 60.0% for PSMA PET/CT (*p* = 0.016), and 80.0% for both modalities in conjunction. Of the four patients with recurrence involving the bladder, participating radiologists had an accuracy of 90.0% with MRI alone, 65.0% with PSMA PET/CT alone, and 75.0% with both imaging modalities. These variations in performance were not statistically significant. For the one lesion < 5 mm, one radiologist detected it on mpMRI, three detected it on PSMA PET/CT, and two detected it using combined imaging. The diagnostic accuracy for detecting lesions with a size of 5 mm to 10 mm was 60.0% with mpMRI, 58.0% with PSMA PET/CT, and 48.0% with both. For lesions greater than 10 mm, the diagnostic accuracy with mpMRI was 83.33%, 71.67% with PSMA PET/CT, and 91.67% with both modalities. The only statistically significant difference seen in our subgroup analysis of different lesions sizes was the improved performance of mpMRI over PSMA PET/CT in lesions greater than 10 mm in size (*p* = 0.031). A comparison of 18 F-piflufolastat and 68Ga–PSMA-11 demonstrated that the diagnostic agent administered did not result in significant changes to diagnostic accuracy for both PSMA PET/CT and combined imaging (PSMA PET/CT: *p* = 0.624, combined imaging: *p* = 0.363). However, while combined imaging resulted in higher accuracy than PSMA PET/CT alone in subjects administered 68Ga-PSMA-11 (80.0% versus 64.3%, *p* = 0.013), it did not in subjects administered 18 F-piflufolastat (*p* = 0.458).

**Table 3:** Comparison of Modality-Based accuracy across subgroups

Subgroup	MpMRI accuracy (%)	PSMA PET/CT accuracy (%)	PSMA PET/CT + mpMRI accuracy (%)
Bladder	90.0 (*p* = 0.063*)	65.00 (*p* = 0.63**)	75.00 (*p* = 0.250***)
Multiple lesions	88.0 (*p* = 0.016*)	60.00 (*p* = 0.063**)	80.00 (*p* = 0.500***)
Lesions < 5 mm	20.0 (*p* = 0.500*)	60.0 (*p* = 1.000**)	40.0 (*p* = 1.000***)
Lesion size 5–10 mm	60.0 (*p* = 1.000*)	58.0 (*p* = 0.227**)	48.0 (*p* = 0.238***)
Lesion size > 10 mm	88.3 (*p* = 0.031*)	71.7 (*p* = 0.002**)	91.7 (*p* = 0.625***)
18 F-piflufolastat agent	73.0 (*p* = 0.543*)	68.0 (*p* = 0.458**)	73.0 (*p* = 1.000***)
68Ga-PSMA-11 agent	72.9 (*p* = 0.307*)	64.3 (*p* = 0.013**)	80.0 (*p* = 0.267***)
Prior radiation therapy	80.0 (*p* = 0.344*)	56.7 (*p* = 0.727**)	76.7 (*p* = 0.688***)

*MpMRI versus PSMA PET/CT p-value, **PSMA PET/CT versus PSMA PET/CT + mpMRI p-value, ***MpMRI versus PSMA PET/CT + mpMRI p-value

### Confidence

On a 1 to 5 scale, the average self-reported confidence when reading studies using mpMRI alone was 3.97 (Table 4). When using PSMA PET/CT alone, that average confidence improved to 4.27 (*p* < 0.001). When using both modalities, average confidence further increased to 4.62 (*p* < 0.001). When utilizing mpMRI, mean confidence was 4.04 for correct diagnoses, while incorrect diagnoses had a mean confidence of 3.79. With PSMA PET/CT, mean confidence was 4.33 for correct diagnoses and 4.14 for incorrect diagnoses. When interpreting imaging of both modalities, the self-reported confidence was 4.69 for correct diagnoses, compared to 4.39 for incorrect diagnoses.

**Table 4:** Radiologist confidence

Modality	Accuracy	Number of scores	Mean confidence
MpMRI	Correct	138	4.04
	Incorrect	47	3.79
	**Overall**	**185**	**3.97 (*p*** < 0.001*)
PSMA PET/CT	Correct	128	4.33
	Incorrect	57	4.14
	**Overall**	**185**	**4.27 (*p*** < 0.001**)
MpMRI + PSMA PET/CT	Correct	144	4.69
	Incorrect	41	4.39
	**Overall**	**185**	**4.62 (*p*** < 0.001***)

*MpMRI versus PSMA PET/CT p-value, **PSMA PET/CT versus PSMA PET/CT + mpMRI p-value, ***MpMRI versus PSMA PET/CT + mpMRI p-value

## Discussion

This study provides insights into the comparative diagnostic performance of mpMRI, PSMA PET/CT, and their combination for detecting local prostate cancer recurrence following radical prostatectomy. Our results demonstrate that mpMRI outperforms PSMA PET/CT across all diagnostic metrics, including sensitivity, specificity, PPV, and NPV. The accuracy of mpMRI (74.59%) was also greater than PSMA PET/CT (69.19%), but this difference was not statistically significant (*p* = 0.275). The superior diagnostic performance of mpMRI can likely be attributed to its utility of both anatomical imaging data (T1WI and T2WI) and functional imaging data (DWI and DCE) [8]. The T2WI provides anatomical orientation and signal pattern evaluation of post-surgical anatomy, while DWI and DCE are able to distinguish recurrent tumors from post-operative changes such as inflammation, fibrosis, or scarring [8].

Our results align with current literature that demonstrates the advantages of MRI in post-prostatectomy patients. For example, one study evaluating 32 subjects with biochemical recurrence found mpMRI had a higher diagnostic accuracy (92.3% vs. 77.8%), sensitivity (90.9% vs. 63.6%), and specificity (94.7% vs. 73.7%) than PSMA PET/CT for detecting local recurrence [16]. In this study, the increase in accuracy with mpMRI was shown to be statically significant. In another study involving 34 post-RP patients at risk of local recurrence, mpMRI identified every case of local recurrence, while PSMA PET/CT only identified 57.7% of cases (*p* = 0.001) [17].

The inferior diagnostic performance of PSMA PET/CT may be attributed to the technical and physiological limitations inherent to PSMA tracer biodistribution. Physiological urinary excretion of PSMA tracers creates a background of high activity that can mask or obscure imaging findings related to recurrent prostate cancer at the locations of the vesicourethral anastomosis, bladder neck, or within the bladder lumen [12]. This limitation was demonstrated in our subgroup analysis of the five patients with lesions involving the bladder, where mpMRI achieved a substantially greater diagnostic accuracy than PSMA PET/CT (90.0% versus 65.0% *p* = 0.063). This highlights the vulnerability of PSMA imaging when surveilling for anastomotic and peri-vesical recurrence. Further, the relatively low sensitivity of 65.2% with PSMA PET/CT underlines the potential for false negative readings. These false negatives may result from tumors with inherently low PSMA expression and are more common in patients with low serum PSA values [18]. A clinical trial of post-RP subjects with BCR has shown that while PSMA PET/CT has an 85.9% detection rate in subjects with a median PSA of 1.99 ng/mL, this rate drops to 57.9% when the PSA is < 0.5 ng/mL [19]. This is relevant to our cohort, in which 15 (40.54%) of subjects had PSA levels below 0.5 ng/mL at the time of imaging. However, it is important to note that the detection of local recurrence with mpMRI has also been shown to improve in patients with higher PSA levels [20]. The choice of PSMA ligand may also influence diagnostic performance, particularly for lesions near or involving the bladder. While 68Ga-labeled tracers are associated with high urinary bladder uptake due to renal excretion, 18 F-labeled tracers typically demonstrate lower bladder activity, potentially improving visualization of anastomotic and peri-vesical recurrence [21, 22]. In our cohort, 14 patients received 68Ga-PSMA-11 while 23 received various 18 F-labeled tracers. Although our subgroup analysis did not reveal significant differences in overall diagnostic accuracy between tracer types, the superior performance of combined imaging over PSMA PET/CT alone was only observed in patients who received 68Ga-PSMA-11 (80.0% versus 64.3%, *p* = 0.013), suggesting that the complementary anatomical detail from mpMRI may be particularly valuable when using 68Ga-labeled tracers with higher bladder interference.

Our study also evaluated the diagnostic performance of combined PSMA PET/CT and mpMRI imaging. Amongst all three imaging techniques (mpMRI, PSMA PET/CT, PSMA PET/CT + mpMRI), the combined modality had the highest specificity (90.0%), PPV (92.0%), NPV (64.9%), and diagnostic accuracy (77.8%). This diagnostic accuracy represented a statistically significant improvement over PSMA PET/CT alone (*p* = 0.026). The specificity increased substantially when mpMRI and PSMA PET/CT were used in combination, demonstrating that concurrent interpretation with both modalities enhances radiologists’ ability to distinguish true local recurrence from false positive findings. Further, both inter-reader and intra-reader agreement was higher when both modalities were employed together. This complementary approach can help mitigate limitations inherent to each individual modality. Specifically, a simultaneous interpretation of mpMRI’s anatomical detail may help radiologists to discriminate the urinary excretion of PSMA tracer from local recurrence [12]. PSMA PET/CTs molecular specificity can help differentiate non-specific enhancements on mpMRI due to post-operative changes from tumor recurrence [23].

**Fig. 2:**
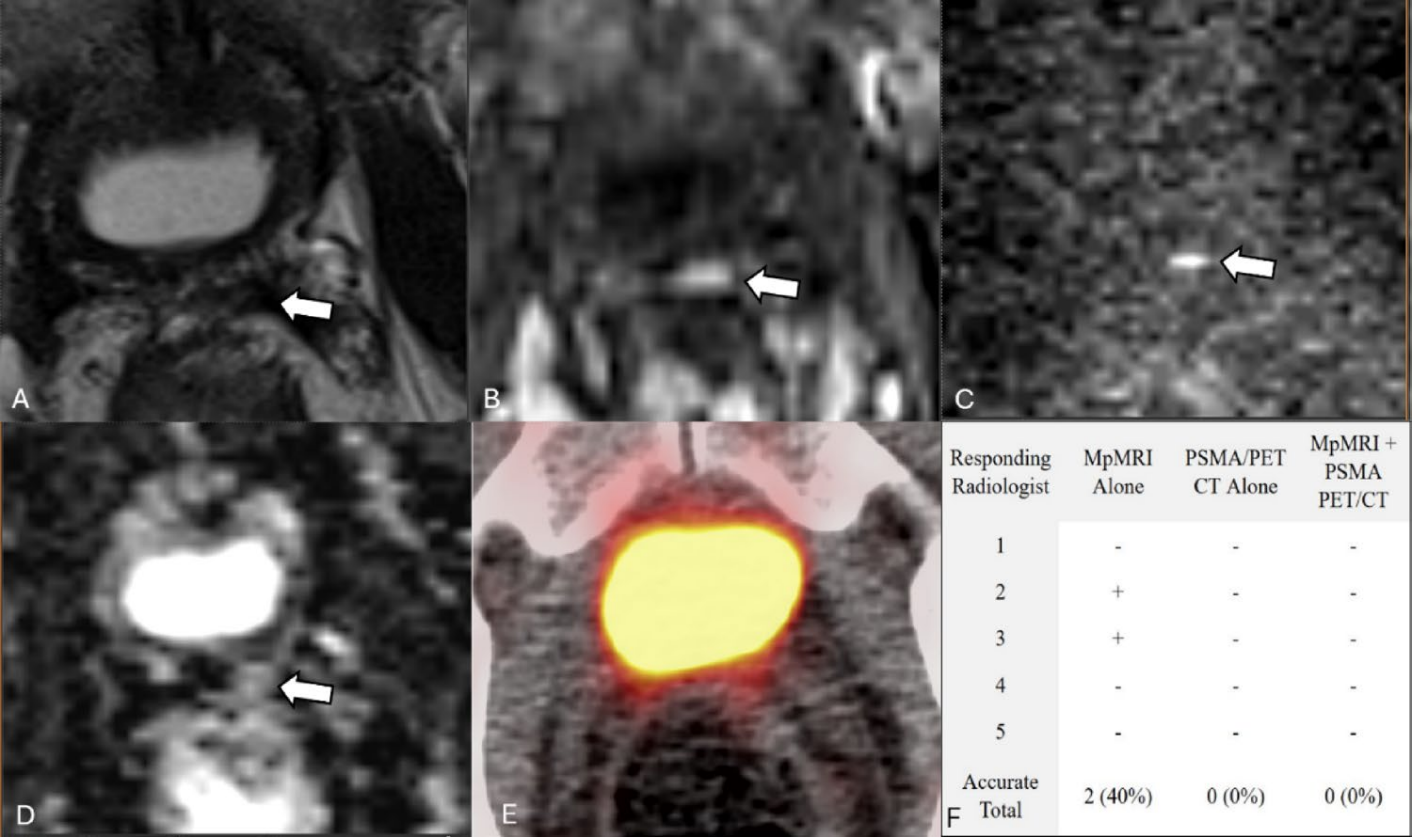
78-year-old patient with a left prostate bed lesion with early enhancement and restricted diffusion on mpMRI but no focal uptake on PSMA PET/CT. This case depicts a 78-year-old status-post prostatectomy indicated for a primary adenocarcinoma with a Gleason score of 4 + 4 = 8 with a T3b pathological stage. This patient also received salvage radiation treatment before imaging. His PSA was measured to be 2.4 ng/mL. MpMRI (**A** T2, **B** Arterial enhancement T1, **C** DWI, **D** ADC) shows a 5–10 mm T2 dark lesion with early enhancement and restricted diffusion in the left prostate bed (white arrow). Gallium-Ga 68 PSMA-11 PET/CT (**E**) shows no focal uptake in this region. MpMRI and PSMA PET/CT were performed 3 days apart. Radiologist performance worsened with both imaging modalities in conjunction compared to mpMRI alone (**F**)

**Fig. 3:**
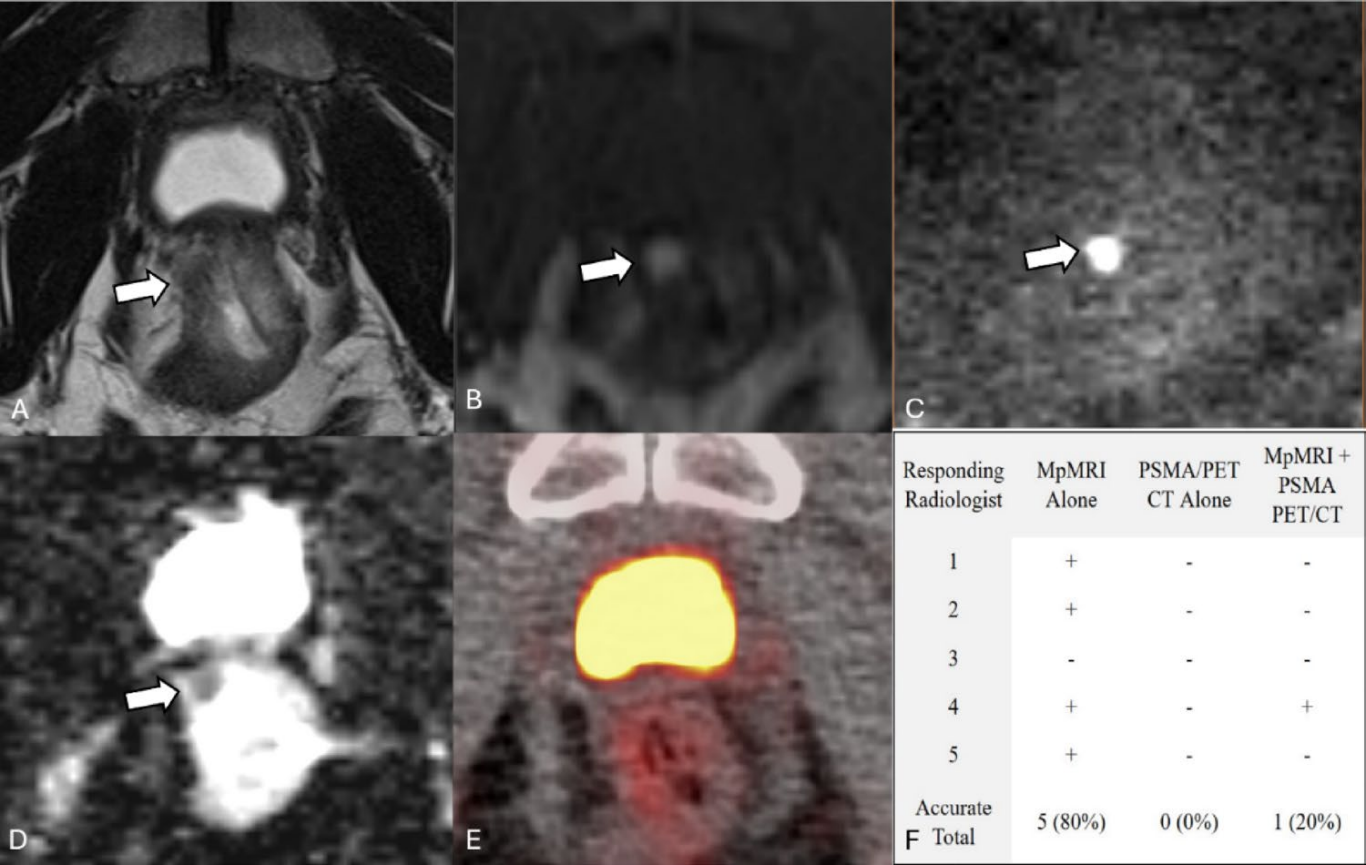
57-year-old patient with a right anterior rectum lesion with early enhancement and restricted diffusion on mpMRI but no focal uptake on PSMA PET/CT. This case depicts a 57-year-old status-post prostatectomy indicated for a primary adenocarcinoma with a Gleason score of 5 + 4 = 9 with a T3b pathological stage. His PSA was measured to be 0.4 ng/mL at the time of imaging. MpMRI (**A** T2, **B** Arterial enhancement T1, **C** DWI, **D** ADC) shows a 5–10 mm T2 dark lesion with early enhancement and restricted diffusion in the right anterior rectum (white arrow). 18 F-piflufolastat PET/CT (**E**) shows no focal uptake above background in this region. MpMRI and PSMA PET/CT were performed 23 days apart. MpMRI alone yielded a higher diagnostic accuracy than the combination of mpMRI and PSMA PET/CT (**F**)

However, our results highlight some notable pitfalls with the joint imaging sequence. Figure 2 depicts a case in which two radiologists correctly identified recurrence on mpMRI, yet all five incorrectly classified as negative on PSMA PET/CT. When interpreting PSMA PET/CT alongside mpMRI, all five radiologists maintained their negative assessment. This pattern highlights a potential overconfidence in PSMA PET/CT, likely due to a high perceived diagnostic specificity. This is supported by higher self-reported confidence when using PSMA PET/CT compared to mpMRI (4.27 versus 3.97, *p* < 0.001). However, given the reported 73.7% specificity of PSMA PET/CT in identifying local recurrence in post-RP patients, radiologists should recognize the potential for false positive findings [16]. Figure 3 provides additional evidence: after only one radiologist failed to identify local recurrence on mpMRI in this case, four missed the recurrence when using both modalities. Radiologists performing post-prostatectomy surveillance for local recurrence should avoid allowing negative PSMA PET/CT findings to override positive mpMRI findings. This is particularly important in cases involving bladder-adjacent lesions or multifocal disease, where our results demonstrate that combined interpretation reduces detection accuracy compared to mpMRI alone.

**Fig. 4:**
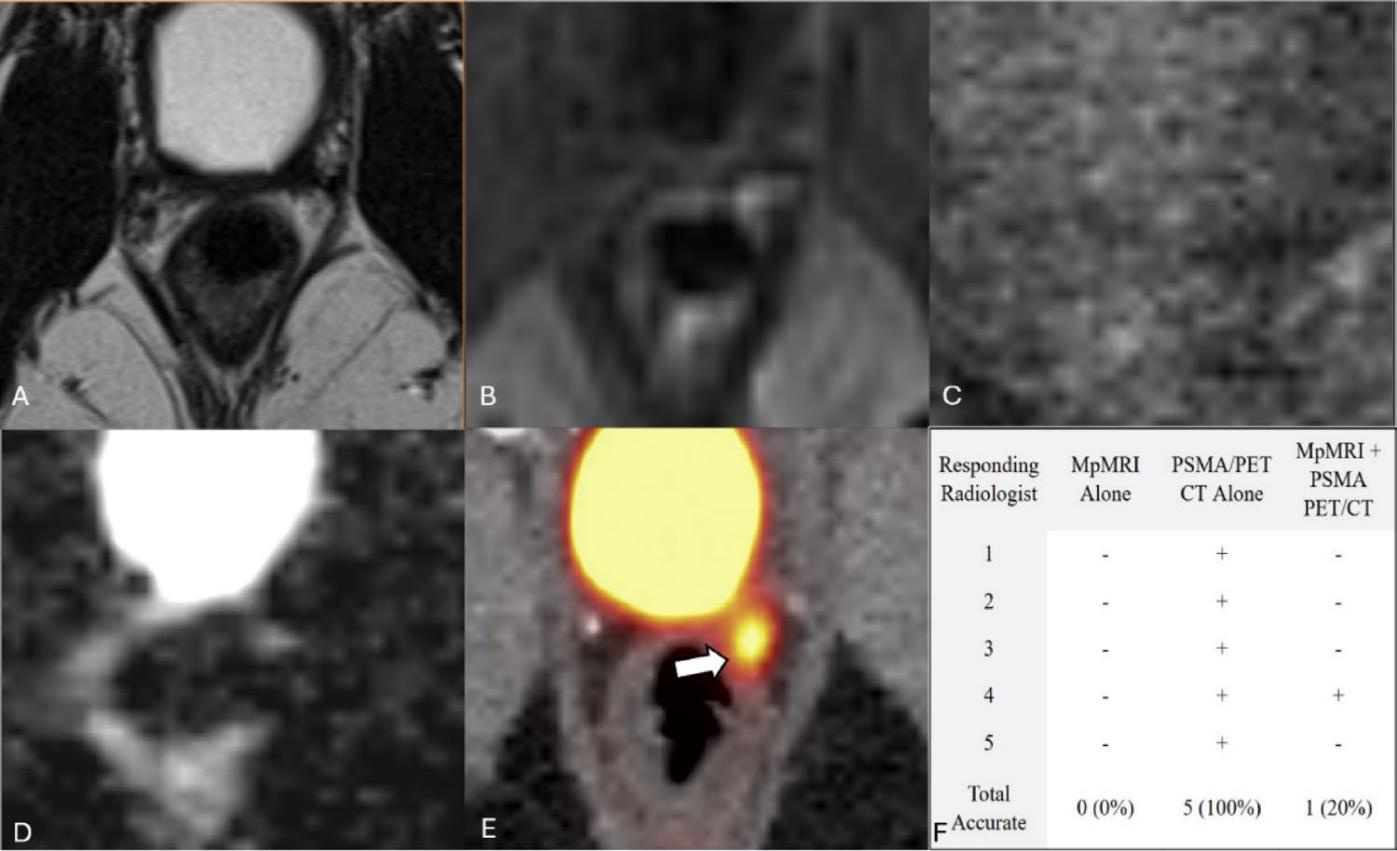
64-year-old patient with a left prostate bed lesion with focal uptake on PSMA PET/CT but no focal abnormalities on mpMRI. This case depicts a 64-year-old status-post prostatectomy indicated for a primary adenocarcinoma with a Gleason score of 4 + 3 = 7 with a T2b pathological stage. His PSA was measured to be 1.0 ng/mL at the time of imaging. MpMRI (**A** T2, **B** Arterial enhancement T1, **C** DWI, **D** ADC) shows no focal MRI abnormalities, but 18 F-piflufolastat PSMA PET/CT (**E**) shows a 5–10 mm area of focal uptake on in the left prostate bed (white arrow). MpMRI and PSMA PET/CT were performed 22 days apart. In this case, performance decreased with the combination of mpMRI and PSMA PET/CT when compared to PSMA PET/CT alone (**F**)

This interference between modalities was not unidirectional. Figure 4 illustrates a case in which all five radiologists correctly identified recurrence on PSMA PET/CT after missing it on mpMRI. However, four radiologists subsequently missed the recurrence when reviewing both modalities in conjunction. Average self-reported confidence for incorrect diagnoses with both modalities together (4.39) was greater than average confidence for correct answers with both modalities individually (mpMRI: 4.03, PSMA PET/CT: 4.33). Although utilizing both modalities improves overall confidence and diagnostic accuracy, radiologists should avoid allowing negative findings from one modality to overshadow or negate positive findings from the other.

While our study suggests that joint interpretation improves overall diagnostic accuracy as well as inter-rater reliability, these interpretive pitfalls represent only one consideration when deciding whether to employ PSMA PET/CT alongside mpMRI. First, the increase in accuracy with both modalities was not statistically significant when compared to mpMRI alone (77.84% versus 74.59%, *p* = 0.25). Further, the sensitivity seen with mpMRI alone was greater than the sensitivity seen when used in conjunction with PSMA PET/CT. Future research should further compare the diagnostic performance in identifying local recurrence with the addition of PSMA PET/CT in larger cohorts of post-prostatectomy patients to validate these findings. Additionally, other factors such as cost and patient burden must also be incorporated into this clinical decision. While our results and current literature suggest post-prostatectomy patients should be evaluated for local recurrence with mpMRI, the addition of the relatively expensive PSMA PET/CT causes added costs, consumes patient time, and introduces additional radiation exposure [24, 25]. These considerations are particularly relevant given the requirement for ongoing surveillance scans. Future research should explore both the cost-effectiveness and patient impact of the addition of PSMA PET/CT in this setting. An alternative approach that warrants consideration is integrated PSMA PET/MRI, which combines functional and anatomical imaging in a single examination. This hybrid modality could potentially mitigate the interpretive pitfalls observed in our study by providing simultaneous molecular and anatomical information, while also reducing radiation exposure and eliminating temporal separation between modalities. However, PET/MRI availability remains limited, and its diagnostic performance for post-prostatectomy local recurrence detection requires further investigation [26].

Our study has several key limitations. First, given not all patients received histopathologic confirmation of local recurrence, a multidisciplinary tumor board consensus was used as the reference standard to evaluate radiologist performance. Although this introduces potential misclassification bias, this approach is necessary because recurrences are often too small to be reliably detected and sampled by transrectal ultrasound-guided biopsy [15]. Prospective studies incorporating treatment outcomes would provide valuable validation of imaging accuracy beyond multidisciplinary consensus review. Second, this study was conducted at a single institution with a relatively small cohort consisting of only 23 positive and 14 negative cases. While this limits the generalizability of our results to more diverse populations, single-institution studies offer the advantage of more standardized imaging protocols, consistent equipment, and uniform interpretation practices. Finally, the retrospective design resulted in natural variation in clinical circumstances across subjects, including time since prostatectomy, varying PSA levels, time between imaging, imaging order, and diagnostic agent material, among others.

## Conclusions

In the surveillance of post-prostatectomy patients with biochemical recurrence, the combined use of PSMA PET/CT and mpMRI enhances the detection of local recurrence. The joint modality yields the highest diagnostic accuracy, specificity, PPV, NPV, and inter-rater reliability. However, the joint modality may lead to misdiagnoses when radiologists allow the negative findings from one modality to obscure the positive findings from the other. Radiologists performing post-RP surveillance should have a thorough understanding of the individual performance characteristics and limitations of both PSMA PET/CT and mpMRI to optimize diagnostic accuracy and avoid interpretive pitfalls. Future research should investigate the cost, clinical impact, and broader implications of incorporating PSMA PET/CT alongside mpMRI in this setting.

## Data Availability

No datasets were generated or analysed during the current study.
